# What Does AMH Tell Us in Pediatric Disorders of Sex Development?

**DOI:** 10.3389/fendo.2020.00619

**Published:** 2020-09-08

**Authors:** Nathalie Josso, Rodolfo A. Rey

**Affiliations:** ^1^Centre de Recherche Saint-Antoine (CRSA), INSERM UMR_S938, Sorbonne Université, Paris, France; ^2^Centro de Investigaciones Endocrinológicas “Dr. César Bergadá” (CEDIE), CONICET-FEI-División de Endocrinología, Hospital de Niños Ricardo Gutiérrez, Buenos Aires, Argentina

**Keywords:** testis, ovary, Turner syndrome, Klinefelter syndrome, persistent Müllerian duct syndrome, gonadal dysgenesis, Sertoli cell, Leydig cell

## Abstract

Disorders of sex development (DSD) are conditions where genetic, gonadal, and/or internal/external genital sexes are discordant. In many cases, serum testosterone determination is insufficient for the differential diagnosis. Anti-Müllerian hormone (AMH), a glycoprotein hormone produced in large amounts by immature testicular Sertoli cells, may be an extremely helpful parameter. In undervirilized 46,XY DSD, AMH is low in gonadal dysgenesis while it is normal or high in androgen insensitivity and androgen synthesis defects. Virilization of a 46,XX newborn indicates androgen action during fetal development, either from testicular tissue or from the adrenals or placenta. Recognizing congenital adrenal hyperplasia is usually quite easy, but other conditions may be more difficult to identify. In 46,XX newborns, serum AMH measurement can easily detect the existence of testicular tissue, leading to the diagnosis of ovotesticular DSD. In sex chromosomal DSD, where the gonads are more or less dysgenetic, AMH levels are indicative of the amount of functioning testicular tissue. Finally, in boys with a persistent Müllerian duct syndrome, undetectable or very low serum AMH suggests a mutation of the AMH gene, whereas normal AMH levels orient toward a mutation of the AMH receptor.

## Introduction

DSD can be defined as conditions where genetic, gonadal, and/or internal/external genital sex are discordant. The most common cause of DSD, congenital adrenal hyperplasia (CAH), is easily recognized, but other conditions may be more difficult to diagnose. Anti-Müllerian hormone (AMH), produced in large amounts exclusively by the fetal and prepubertal testis, is an important parameter for differential diagnosis, particularly in children. AMH plays a key role in male sex differentiation.

## Differentiation of The Genital Tract

Initially, the internal reproductive tract is identical in XX and XY embryos. Mesonephric (Wolffian) ducts form in the intermediate mesoderm during the 4th week and elongate caudally in direction of the urogenital sinus (1). During the 5th week, coelomic cells specified to become Müllerian cells form a cleft between the gonadal and mesonephric ridges, laterally to the Wolffian ducts. Then, these cells invaginate caudally until they reach the Wolffian duct, a step requiring the expression of WNT4 by the mesonephric mesenchyme (2). The Müllerian ducts grow toward the urogenital sinus, crossing the Wolffian ducts ventrally, thus finally lying medially and fusing to give rise to the uterovaginal canal in the midline (3). Elongation of the Müllerian duct is regulated by WNT9B secreted by the Wolffian duct epithelium (4) and requires physical contact with the latter (3).

The subsequent fate of the Müllerian duct differs markedly according to sex. In the normal male, its cranial end shows signs of impending regression even before the Müllerian duct reaches the urogenital sinus, coinciding with the beginning of secretion of AMH by Sertoli cells. The Müllerian duct morphologically resembles an epithelial tube but expresses mesenchymal cell markers. These mesoepithelial characteristics persist during regression while, at the same age, the female Müllerian duct becomes exclusively epithelial, heralding the end of the window of sensitivity to AMH (5). Müllerian regression is characterized by loss of the epithelial basement membrane and by apoptosis, progressing toward the urogenital sinus. In the human fetus at 9 weeks, Müllerian ducts have nearly totally disappeared.

Leydig cells, under the effect of placental human chorionic gonadotropin (hCG), produce testosterone, which acts directly on the Wolffian ducts promoting their differentiation into epididymides, vasa deferentia, and seminal vesicles by the 12th week. Male differentiation of the genitalia, including fusion of the labioscrotal folds to form the scrotum, closure of the urethral folds, and positioning of the meatus at the tip of the phallus, is completed by the end of the first trimester of fetal life. The genital tubercle forms the corpora cavernosa and corpus spongiosum of the penis. In the second half of gestation, fetal pituitary LH takes over the regulation of testosterone production, which drives the increase in penile size and—together with the Leydig cell factor insulin-like 3 (INSL3)—the descent of the testes to scrotal position.

In the absence of testicular hormones, whether ovaries are present or not and irrespective of karyotype, differentiation of the genital tract follows the female pathway. In the absence of AMH, the Müllerian ducts form the Fallopian tube, the uterus, and the upper part of the vagina. They become resistant to AMH when they lose their mesenchymal markers to become purely epithelial (5). In the absence of androgen action, Wolffian ducts regress through an active process induced by COUP-TF2 (6). The prostate does not differentiate, and the vagina opens separately from the urethra on the surface of the perineum. The urethral folds do not fuse and give rise to the labia minora; the labioscrotal swellings also remain separated to form the labia majora. The genital tubercle does not grow and forms the clitoris. For a detailed description of sex differentiation, see ref. (7).

## AMH, A Member of The TGF-Beta Family

AMH, a member of the transforming growth factor beta (TGFβ) family (8) secreted by Sertoli cells immediately after testicular differentiation, is responsible for the regression of Müllerian ducts in the male fetus. Like other members of the TGFβ family, AMH is translated as a dimeric precursor protein comprising two polypeptide chains, each containing a large N-terminal pro-region and a much smaller C-terminal mature domain homologous to those of the other members of the TGFβ family. Proteolytic cleavage at arginine 451 yields 110-kDa N-terminal and 25-kDa C-terminal dimers which remain associated in a bioactive non-covalent complex (9). The human AMH gene (8), only 2.8 kb long and located on chromosome 19p13.3 (10), contains five exons; the 3′ end of the fifth exon encodes the bioactive C-terminal domain. GATA, SF1, and SOX9 binding sites present within 418 bp of the translation start site activate AMH expression; sites further upstream are required for normal regulation (11).

Like other members of the TGFβ family, AMH signals through two membrane-bound serine/threonine kinase receptors and several cytoplasmic R-SMAD effectors. The primary receptor, *AMHR2* gene contains 11 exons spread over 8 kbp and maps to chromosome 12q13.13 (12). After binding of AMHR2 to its specific ligand AMH, the complex recruits a type I receptor, either the BMP receptors BMPR1A, aka ALK3, or BMPR1B, aka ALK6, or the activin receptor ACVR1, aka ALK2, resulting in the phosphorylation of SMADS 1, 5, or 8. The type I receptor and SMAD repertoire is shared with the bone morphogenetic protein family, AMH's closest relatives within the TGFβ family (7).

**Figure 1:**
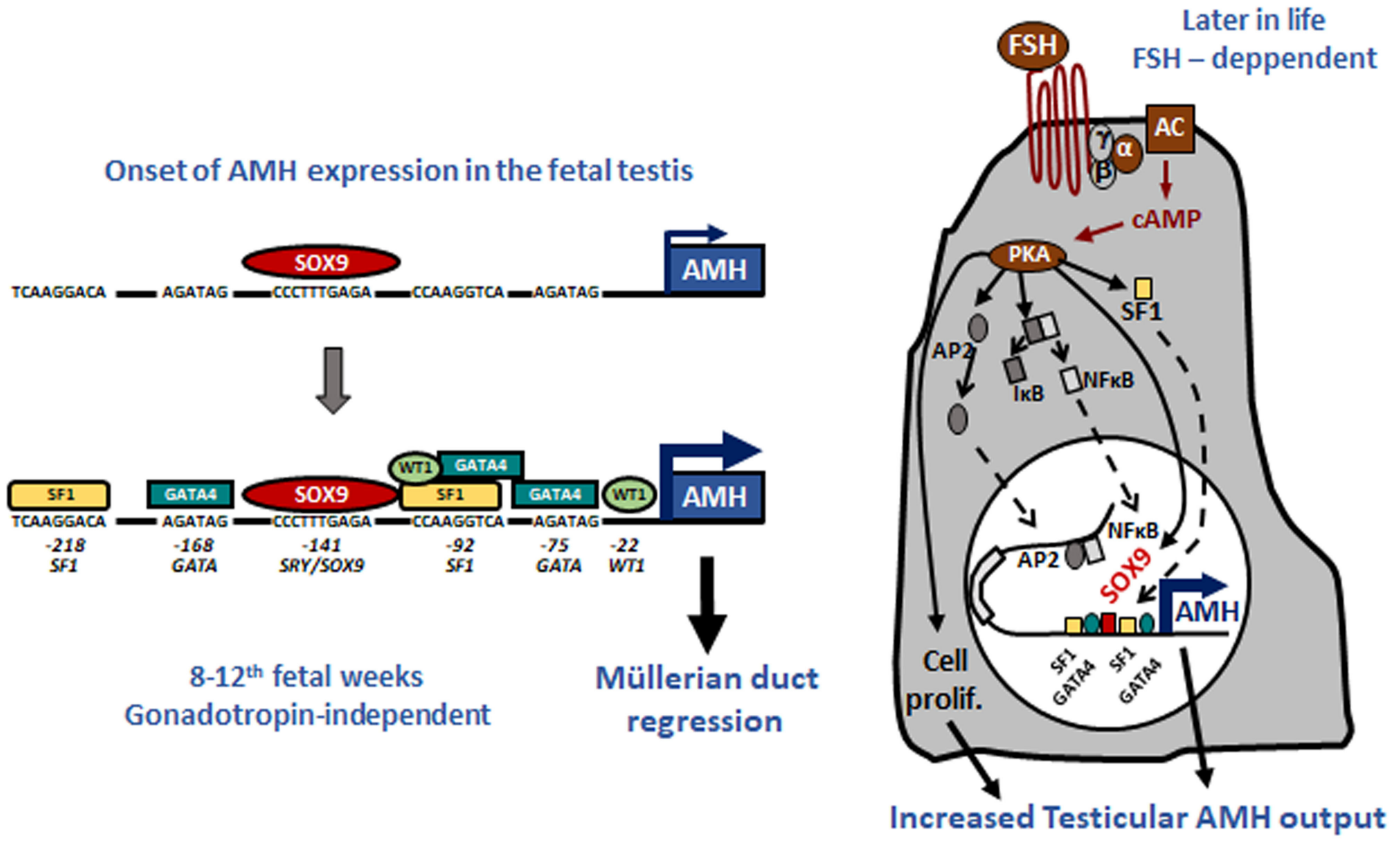
Upregulation of AMH production in testicular Sertoli cells. Left: onset of *AMH* expression in early fetal life is independent of gonadotropins and depends on transcription factors SOX9, which triggers *AMH* expression, and SF1, GATA4, and WT1, which further increase *AMH* transcription by binding to specific response elements on the proximal *AMH* promoter. Right: increase of testicular AMH production in response to FSH, involving the FSH receptor-Gsα protein-adenylate cyclase (AC)-cyclic AMP (cAMP) pathway, which activates protein kinase A (PKA)-mediated induction of SOX9, SF1, NFκB, and AP2. These factors bind to their specific response elements on the *AMH* promoter. Reproduced with permission from ref. (11). Copyright^©^ 2011 the American Physiological Society and ^©^ 2020 MDText.com, Inc.

**Figure 2:**
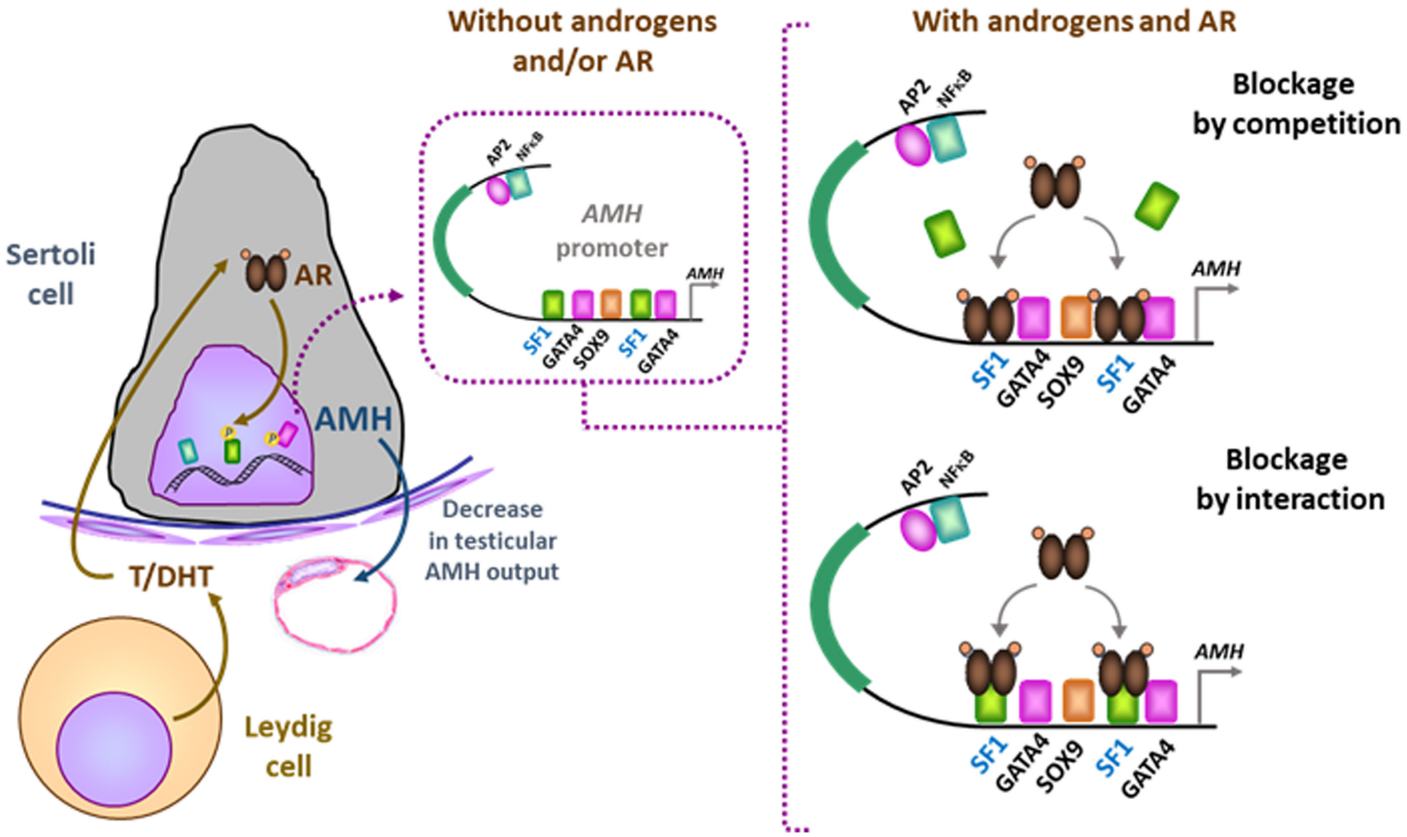
Downregulation of AMH production in testicular Sertoli cells by androgens. In the absence of androgens (e.g., normal childhood and DSD with impaired androgen production) or androgen receptor (AR, e.g., normal fetal and neonatal periods and DSD due to androgen insensitivity syndromes), SF1 binds to its response elements on the *AMH* promoter and increases *AMH* transcription. When androgen action on the AR is effective in the Sertoli cell (e.g., at puberty), the ligand-activated AR could either block SF1 binding to the *AMH* promoter (blockage by competition) or interact with site-bound-SF1 and prevent it from exerting its stimulatory effect on *AMH* promoter (blockage by interaction). Modified with permission from (17). Copyright ^©^ The Author(s) 2018.

Tight regulation of AMH transcription is crucial for regression of Müllerian ducts since they are able to respond to AMH only within a very narrow time window (13). Surprisingly, however, production does not cease once Müllerian ducts have disappeared; it continues up to puberty. Initially, transcription requires the cooperation of various transcription factors, SOX9, SF1, GATA4, and WT1, to name only a few [reviewed in (14)]. Later in fetal life and after birth, testicular AMH production is increased by FSH signaling through its seven-transmembrane receptor and the pathway involving the Gsα subunit (15), cyclic AMP, protein kinase A, and transcription factors SOX9, SF1, AP2, and NFκB (11) (Figure 1). Transcription is downregulated by androgens (16). Testosterone acts through the androgen receptor and requires intact binding sites for SF1 on the AMH promoter (17) (Figure 2).

## Why Measure AMH In DSD?

Müllerian ducts have completely disappeared in the male 10 weeks after conception, but testes continue to churn out high amounts of AMH throughout childhood, when basal testosterone and gonadotropin levels have little clinical use. It is precisely the fact that AMH continues to be secreted at high levels by Sertoli cells during infancy and childhood, when it has no longer a physiological action on Müllerian ducts, which makes AMH such an appealing biomarker for pediatric endocrinologists, not to mention that prior gonadotropin stimulation is not required. In theory, to determine whether AMH has been secreted or not in a DSD patient, a look at Müllerian derivatives by laparoscopy or sonography should suffice. However, laparoscopy is invasive, and sonography is not always reliable in newborns. Measurement of AMH concentration in serum by ELISA has been available since 1990 and is now offered by multiple companies (please refer to another article devoted to AMH assays in the same series). However, the information provided by imaging and AMH measurement is not similar. The state of the Müllerian derivatives reflects the effect of AMH secreted very early in fetal life, while serum AMH reflects the amount secreted at the time blood is drawn, and the two do not necessarily coincide (18).

**Figure 3:**
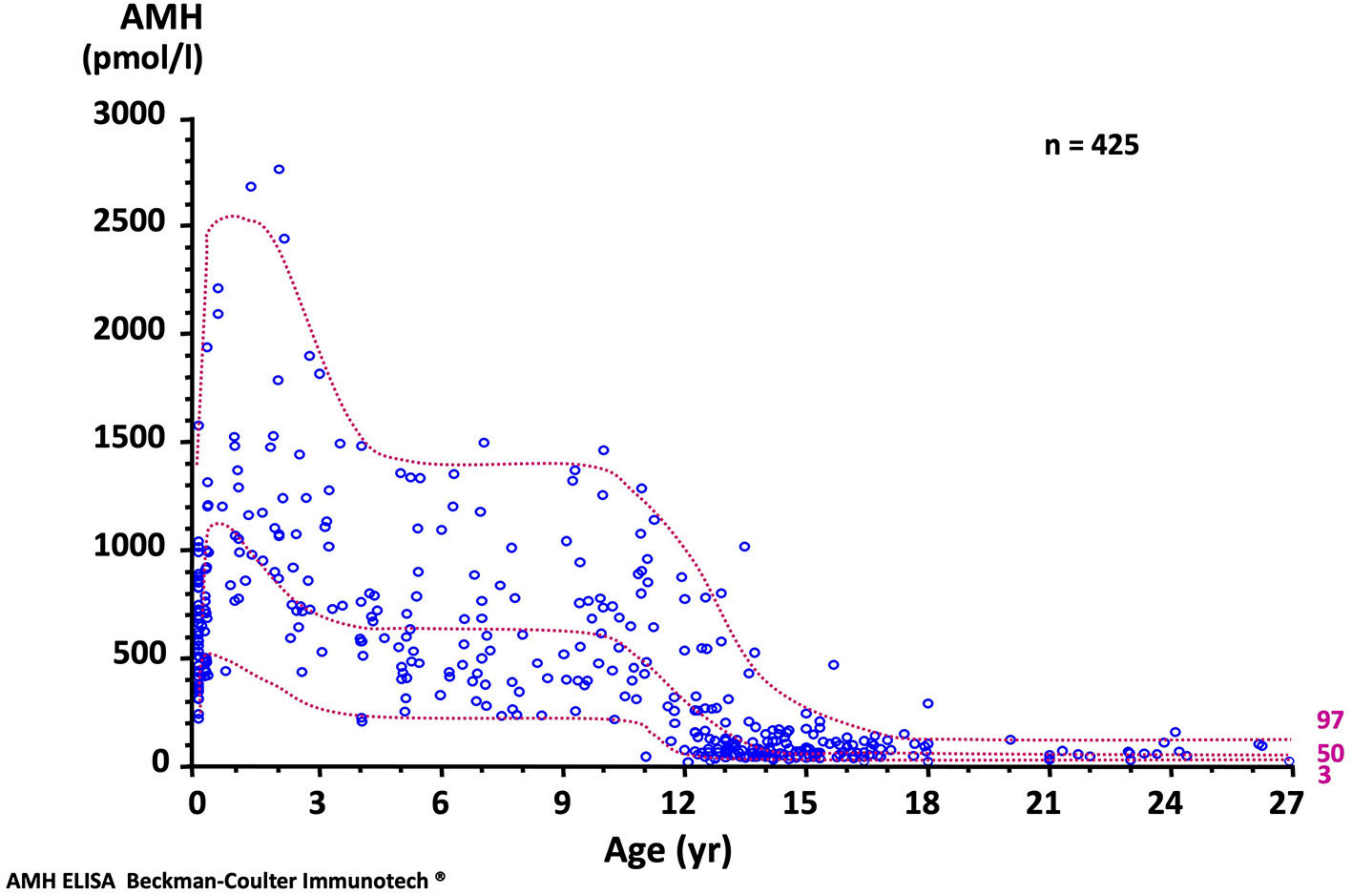
Serum AMH levels in normal males. The dotted lines represent the 97, 50, and 3rd percentiles. To obtain serum AMH in ng/mL, divide by 7.14. Reproduced with permission from ref. (22). Copyright ^©^ The Author(s) 2011.

The concentration of AMH, one of the first proteins produced by the fetal testis, is high in serum during fetal life though not detectable in amniotic fluid (19). Although it declines transiently at birth, it remains distinctly higher in males than in females and increases again during the 1st month (20) to reach its peak in the 2nd year of life (21, 22). Circulating levels remain high during childhood but fall at the onset of puberty, downregulated by the rising intratesticular concentration of testosterone. The normal AMH serum concentration in developing boys is shown in Figure 3. Interestingly, during the fetal period and early infancy, Sertoli cells do not express the androgen receptor and are thus insensitive to the high levels of testosterone of minipuberty (23–25) and references therein.

What does AMH tell us in DSD? Serum concentrations clearly above female values indicate that testicular tissue is present. Testicular tissue virilizes the fetus through the combined action of two hormones, AMH and testosterone. If both are defective, global testicular dysgenesis is probably involved. If only one is deficient, a block in the synthesis or action of either testosterone or AMH is likely. Serum AMH is also a reliable biomarker of the balance between FSH and androgen action within the testis (26): high AMH indicates FSH stimulation and lack of androgen action as in androgen insensitivity whereas low AMH suggests a predominant androgen inhibiting action, as in precocious puberty. Furthermore, in prepubertal DSD patients, serum AMH measurement monitors Sertoli cell function, a very useful feature since testicular tissue tends to deteriorate over time in DSD.

In the ovary, low amounts of AMH are produced by the granulosa cells of primary and small antral follicles (27–29), starting from the 25th week of fetal life (30). At that time, Müllerian derivatives are no longer sensitive to AMH (5, 31). Serum AMH is 50-fold lower in girls than in boys at birth (20) and remains relatively stable from childhood through young adulthood (32).

## The 46,XY Child With DSD

**Figure 4:**
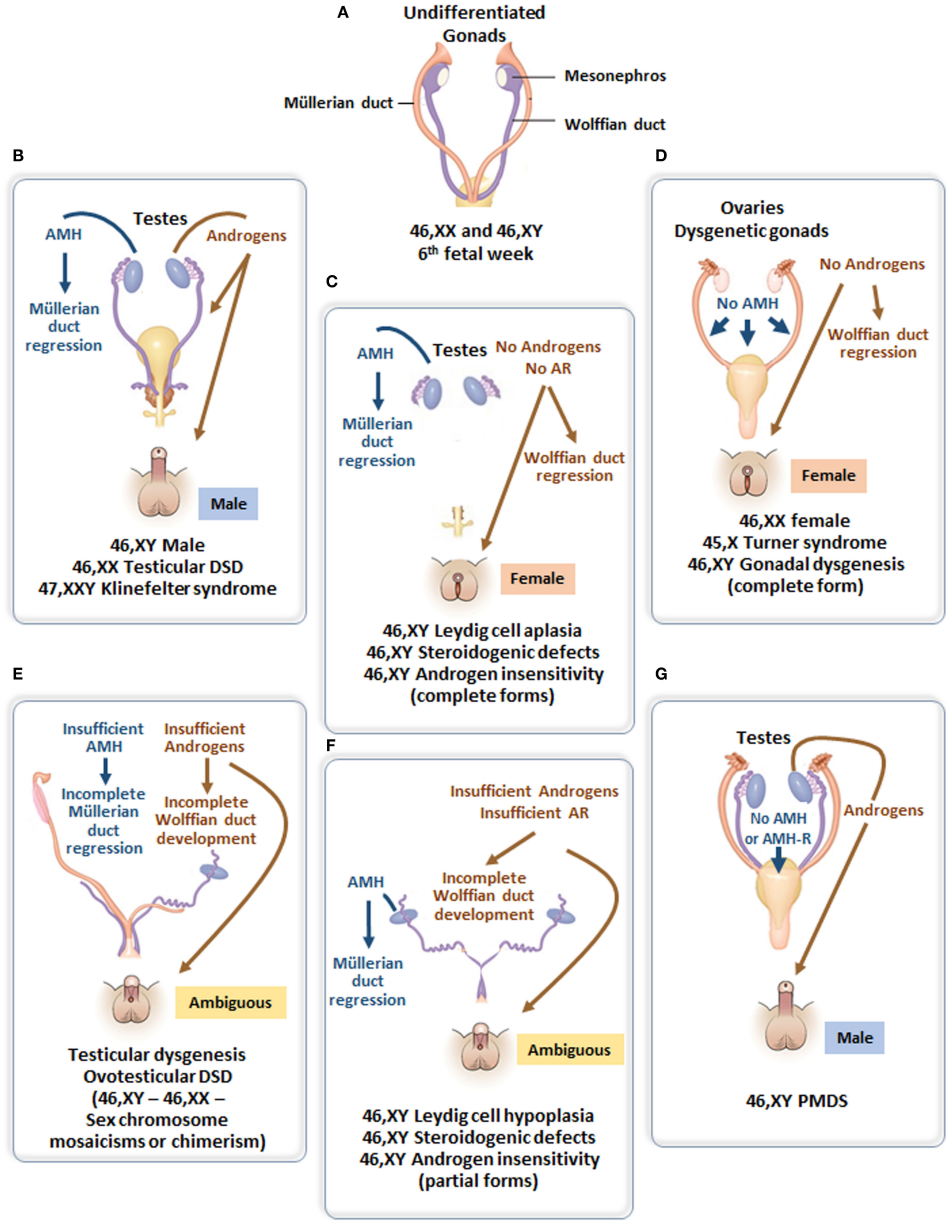
**(A)** Undifferentiated stage of fetal sex development. **(B)** Male differentiation in normal 46,XY individuals and patients with 46,XX testicular DSD or Klinefelter syndrome. **(C)** Female external genitalia in patients with 46,XY DSD due to impaired androgen synthesis or action. **(D)** Female genital differentiation in normal 46,XX individuals and patients with dysgenetic DSD associated with 46,XX, 45,X or 46,XY genotypes. **(E)** Ambiguous external genitalia in patients with testicular or ovotesticular dysgenesis with different karyotypes. **(F)** Ambiguous external genitalia in patients with 46,XY DSD due to impaired androgen synthesis or action. **(G)** Male external genitalia and persistence of Müllerian ducts in 46,XY patients with *AMH* or *AMHR2* gene defects.

According to the original DSD consensus statement (33) updated in 2016 (34), DSD are initially classified according to the patient's karyotype into 46,XY, 46,XX, and sex-chromosome DSD (Table 1). Incomplete or total lack of virilization of the external genitalia in 46,XY individuals may result from insufficient testosterone production by the gonads or defective androgen action at the target organ level. If the deficiency is complete, genitalia have a normal female appearance at birth (Figure 4), and the condition may go undiagnosed until puberty, unless a karyotype is performed for other reasons, before or after birth. Partial defects result in genital ambiguity leading to earlier medical intervention.

**Table 1:** Classification of disorders of sex development (DSD) according to the karyotype and the underlying pathogenesis.

**Affected process**	**46,XY**	**46,XX**	**Chromosomal**
Gonadal differentiation	Complete (or Pure) gonadal dysgenesis Partial testicular dysgenesis	XX male Ovotesticular DSD	Asymmetric gonadal differentiation (or mixed gonadal dysgenesis) Ovotesticular DSD Klinefelter and Turner syndromes
Androgen production (isolated)	Leydig cell aplasia/hypoplasia Steroidogenic defects	Congenital adrenal hyperplasia Aromatase deficiency Exposure to maternal androgenic tumors or drugs	None
Androgen action (isolated)	CAIS / PAIS	None	None
AMH production or action (isolated)	PMDS	None	None

*CAIS, Complete androgen insensitivity syndrome; PAIS, Partial androgen insensitivity syndrome; PMDS, persistent Müllerian duct syndrome*.

### Combined AMH and Testosterone Insufficiency: A Hallmark of Gonadal Dysgenesis

Testosterone, a steroid responsible for genital virilization, and AMH, a glycoprotein member of the TGFβ family, are entirely different molecules with specific biosynthetic and signaling pathways. A defect in both implies that the entire testis is abnormal. In the most extreme scenario, genitalia are completely female and Müllerian derivatives; uterus and Fallopian tubes are normal, suggesting that testes have never existed or functioned in the first place (Figure 4D). Serum AMH is undetectable (35, 36) (i.e., even lower than in 46,XX girls). This condition, known as **pure (or complete) gonadal dysgenesis** or Swyer syndrome, is the complete form of early fetal-onset primary gonadal failure (37, 38) and is sometimes labeled “sex reversal” although gonads are represented by streaks, not ovaries.

#### Primary Gonadal Failure May Be Partial

The external genitalia are partially virilized, resulting in micropenis and hypospadias or clitoral hypertrophy and labial fusion in a female perspective. Ultrasound examination usually reveals the presence of more or less developed Müllerian derivatives (Figure 4E). The serum AMH concentration is lower than in boys of similar age. Testosterone response to hCG is also blunted, but this in itself does not rule out the possibility of an isolated defect in Leydig cell steroidogenesis or a mutation in the LH/CG receptor. However, in the latter conditions, AMH is produced normally, there are no Müllerian derivatives, and AMH concentration is normal for age and sex. A frequent variant of partial gonadal failure is represented by mixed or asymmetrical gonadal dysgenesis (39), characterized by the presence of a testis on one side and a streak gonad on the other (40). These patients often exhibit congenital malformations evocative of Turner syndrome, and their karyotype is typically 45,X/46,XY. Müllerian derivatives are often absent on the testicular side. Familial cases of complete or incomplete gonadal dysgenesis are frequent. Molecular defects affecting the testis determination pathway (41) are often detected. In contrast to autosomal recessive steroidogenic defects, gene haploinsufficiency is often sufficient to disrupt testicular differentiation.

All patients with XY gonadal dysgenesis are at risk for malignant germ cell tumors and should be closely monitored (42). In itself, presence of Müllerian remnants should alert the clinician to this possibility. Formerly, cancer risk was a decisive argument for choosing a female sex of rearing in poorly virilized patients with Müllerian organs, because the decision involved early bilateral gonadectomy. Today, some human rights activists consider that genitoplasty of infants should be outlawed [see (43–45)], with the result that dysgenetic testes may be left *in situ* until the child reaches prepuberty (46), a particularly dangerous age for the onset of germ cell malignancy (47).

### Isolated Testosterone Insufficiency: Steroidogenesis Defect or Testosterone Insensitivity?

AMH is also helpful if an androgen synthesis or action defect is suspected in a patient with DSD. Indeed, ambiguous or female genitalia are indicative of androgen failure, but testosterone levels cannot distinguish between gonadal dysgenesis and specific Leydig cell disorders or androgen insensitivity during childhood. Serum AMH can orient the diagnosis: it is low or undetectable in gonadal dysgenesis, but normal or high in isolated Leydig cell disorders or androgen insensitivity.

#### Testosterone Synthesis Defects

**Figure 5:**
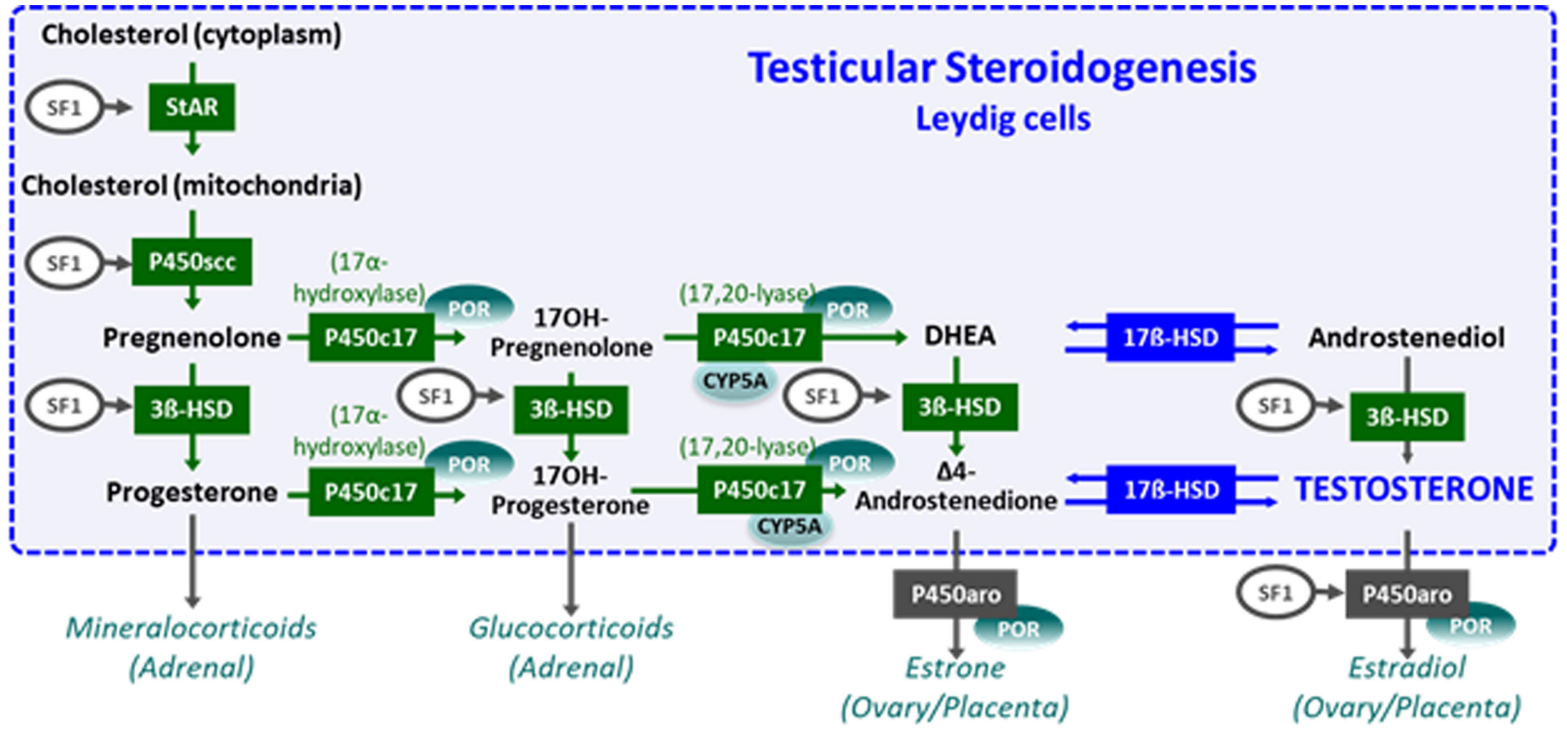
Steroidogenesis steps in testicular Leydig cells (within the dotted blue box) and in the adrenals, ovaries, and placenta (outside the box). Reproduced with permission from (49). Copyright^©^ 2020 Grinspon, Bergadá and Rey.

The initial steps of steroidogenesis are shared by the adrenals and the gonads; thus, defects of testosterone synthesis are often associated with rare forms of congenital adrenal hyperplasia (48) (Figure 5). Briefly, cholesterol is transferred to the inner mitochondrial membrane by the steroidogenic acute regulatory protein (StAR) in response to LH or hCG and then converted to pregnenolone by the cytochrome P450 side chain cleavage (P450scc), an enzyme located at the inner mitochondrial membrane. 3ß-Hydroxysteroid dehydrogenase (3ß-HSD) and ultimately 17β-hydroxysteroid dehydrogenase (17ß-HSD) sequentially synthesize testosterone from the pregnenolone precursor. P450 oxido-reductase (POR) serves as an electron donor for all microsomal cytochrome P450 enzymes (50). Mutations in any of these enzymes or in the LH/CG receptor curtail hCG-stimulated testosterone production to levels observed in patients with gonadal dysgenesis (51). However, the level of serum AMH is normal or elevated and no Müllerian derivatives are detectable, allowing to make the distinction (Figure 4C). The identification of the defective enzyme requires the assay of steroid hormone precursors prior to Sanger sequencing of the suspect gene unless massive parallel sequencing targeted to DSD genes is chosen as a shortcut. All steroidogenic defects are transmitted as recessive autosomal traits, including those that affect testosterone metabolism.

#### Dihydrotestosterone Synthesis Defects

Testosterone can be converted to its more potent metabolite DHT by the enzyme 5α-reductase type 2 (52) expressed in androgen target organs such as fetal genital skin, male accessory sex glands, and prostate. Mutations in the SR25A2 gene are the most frequent cause (53, 54). Alternatively, DHT can be synthesized through the “backdoor” pathway without going through a testosterone intermediate (55). Defects in DHT production lead to very poor virilization of target organs, and most patients are considered girls at birth. Serum testosterone is unremarkable; the T/DHT ratio is high, after hCG stimulation if necessary. The AMH level is in the lower range of normal (56), indicating that testosterone does not need to be metabolized to DHT to regulate AMH production by the Sertoli cell. From a practical viewpoint, it is very important to distinguish 5α-reductase deficiency from other types of XY DSD because if raised as girls, patients often switch to a male sex at puberty, an unusual occurrence in androgen insensitivity.

**Androgen insensitivity** is the consequence of a mutation in the androgen receptor that is coded by a gene on the X chromosome; transmission is recessive sex-linked, affecting only males who lack a normal X chromosome. Again, the condition can be complete (CAIS), characterized by an external female phenotype or partial (PAIS), leading to ambiguous genitalia. In both instances, Müllerian ducts are normally regressed (Figure 4C). The level of serum AMH depends on the age of the patient. During the 1st year of life and at puberty, AMH levels are extremely high, reflecting the stimulation of AMH by FSH unimpeded by testosterone action (57). Serum testosterone is also elevated. Pubertal maturation is female in CAIS, except that menstruation cannot occur. In children, at present, molecular analysis of the androgen receptor is the diagnostic method of choice since testosterone and gonadotropin levels overlap for PAIS, 5α-reductase type 2 deficiency and steroidogenic defects.

**Figure 6:**
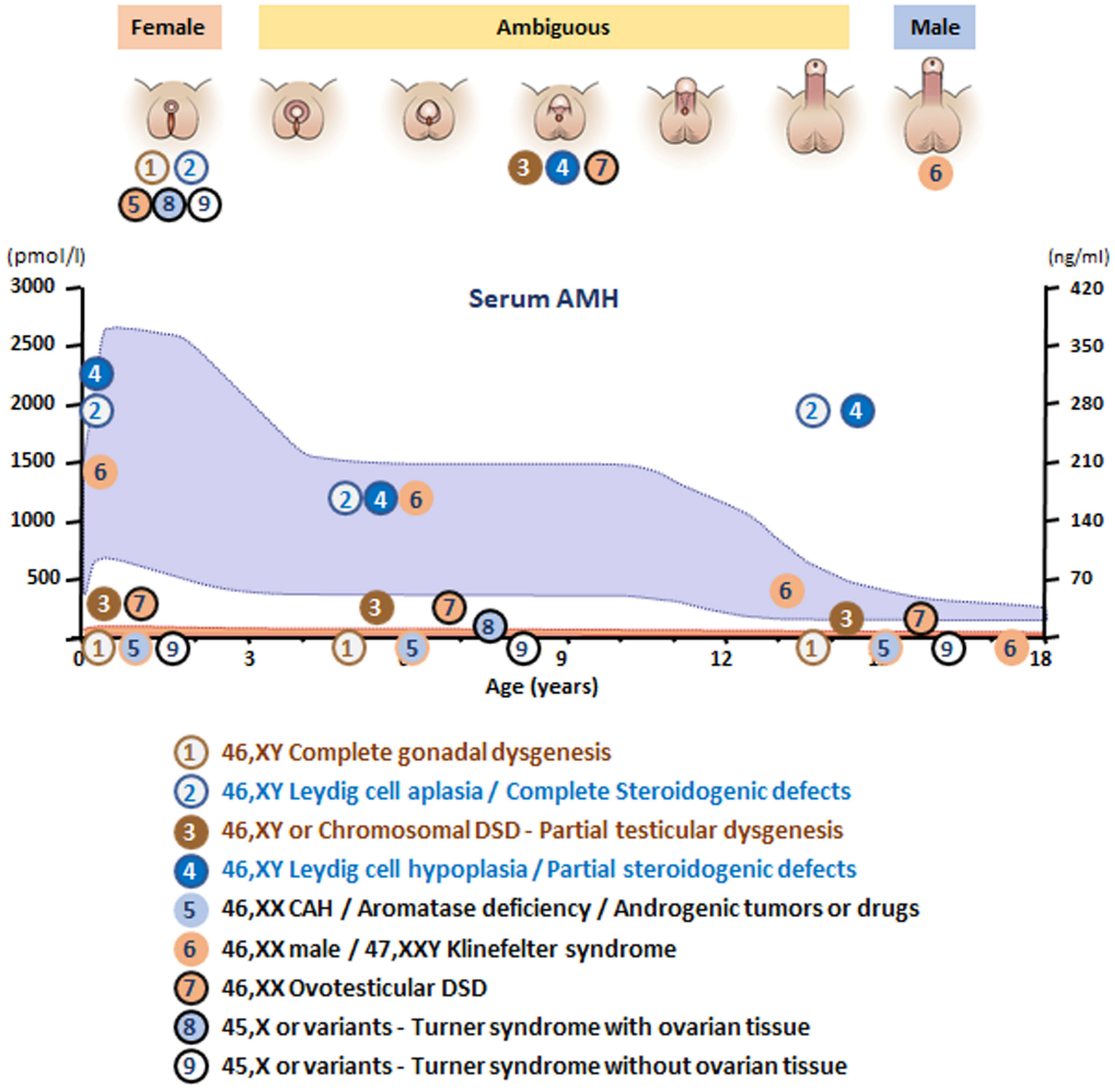
Schematic of AMH levels in various types of disorders of sex development (DSD) in relationship to the aspect of the external genitalia and age. The shaded area represents reference levels for AMH, as obtained from ref. (22).

In summary, in undervirilized XY DSD, low AMH is typical of gonadal dysgenesis (1 and 3 in Figure 6) while normal to high levels are observed in androgen insensitivity and androgen synthesis defects (2 and 4 in Figure 6). In most other forms of XY DSD linked to testosterone insufficiency, the AMH serum level is normal for age and diagnosis rests essentially on the assay of testosterone and its precursors as well as metabolites and gonadotropins. However, biochemical results are not always clear-cut, and today, molecular analysis is assuming an increasingly important role. Targeted or whole exome new-generation sequencing has significantly improved the proportion of diagnosed XY DSD cases (58, 59).

### The Male Child With Impalpable Testes

At first glance, a newborn boy with impalpable testes is not a DSD candidate, unless his karyotype dictates otherwise. Once a 46,XY karyotype has been ascertained, the pediatrician must choose between two possibilities: bilateral cryptorchidism or anorchia. AMH is very helpful in this regard if its level is higher than the normal female value, testicular tissue must exist (60, 61) because the prepubertal testis is the only organ capable of secreting large amounts of AMH.

**Cryptorchidism** is frequently observed in newborns, particularly in preterm ones. To test for the presence of testicular tissue, basal testosterone assay and gonadotropin measurement may not be informative, depending on age. In contrast, an AMH level above female values establishes without a doubt that testicular tissue is present and is an indication for surgery if the testes do not descend spontaneously within the first 6 months of life. AMH is moderately decreased in cryptorchidism; even unilateral but still much higher than in females (62), an hCG test is not required.

In patients with impalpable testes, **anorchia**, although rare, is a possibility that should be considered. Since the external and internal phenotype is unequivocally male, it is obvious that testes were present at least up to month 4 of fetal life and disappeared after fetal sex differentiation was completed. Indeed, in some instances, testes were palpable at birth and vanished later. The condition is apparently due to degeneration subsequent to testicular torsion, and no genetic alterations were detected in 26 cases (63). AMH is not detectable in serum; however, ultrasound examination should be performed to rule out persistent Müllerian duct syndrome (PMDS) due to an AMH mutation. Testosterone and gonadotropin assays are also helpful in this regard.

### Isolated AMH Insufficiency: The Persistent Müllerian Duct Syndrome

Most types of DSD are characterized by testosterone dysfunction, isolated or not. The persistent Müllerian duct syndrome (PMDS) is the only example of DSD due to an isolated defect of AMH synthesis or action (Figure 4G). PMDS is a rare autosomal recessive disorder characterized by the persistence of Müllerian derivatives, uterus, and Fallopian tubes, in otherwise completely virilized 46,XY males. Bilateral cryptorchidism is observed most frequently: the uterus remains anchored to the pelvis and mechanically prevents testicular descent because it is tethered to the testes by the male excretory ducts. Alternatively, one or both testes may make it into the inguinal canal or the scrotum, dragging the uterus along. This may result either in unilateral cryptorchidism with a hernia containing the uterus on the opposite side, a condition known as “*hernia uteri inguinalis*.” The testis on the opposite side can be drawn into the same hemiscrotum by gentle traction or may already be present there; this condition typical of PMDS is named “transverse testicular ectopia.” The anatomical picture may vary within the same family and is not correlated with the genotype (64).

The condition is due to mutations of either the AMH or AMHR2 gene (65). Molecular diagnosis has now been achieved in nearly 200 patients worldwide. Serum AMH is usually very low or undetectable in mutations of the AMH gene, even those affecting the inactive N-terminal proregion. In contrast, the AMH serum level does not significantly differ from control values in mutations of AMHR2 or in idiopathic cases, in which no genetic abnormality of either AMH or AMHR2 has been detected, but this is becoming rarer as new-generation massive parallel sequencing is gaining ground.

In practice, diagnosis of PMDS poses few problems in familial cases or in patients with *hernia uteri inguinalis* or transverse testicular ectopia, both very evocative. In patients with impalpable testes, other disorders must be ruled out such as simple bilateral cryptorchidism, anorchia, or even Prader V congenital adrenal hyperplasia, assuming karyotype analysis has not been performed. Paradoxically, in this regard, AMH assay is of little value because according to the gene involved, serum AMH can be either undetectable as in anorchia and CAH or normal or in the lower range of normal, as in cryptorchidism. Ultrasound pelvic examination to display Müllerian organs is the diagnostic method of choice. AMH assay is useful only to orient molecular investigation.

## The 46,XX Child With DSD

Complete or partial virilization of an XX individual is due to the production of androgen, either from testicular tissue or from other sources: the fetal adrenal, the placenta, or very rarely an ovarian tumor in the mother. Adrenals, placenta, and ovaries do not synthesize AMH, at least not in significant amounts. Thus, AMH assay can easily detect the presence of testicular tissue in such patients.

### 46,XX DSD With Testicular Tissue

Testes are not expected to differentiate in the absence of a Y chromosome, with possible exceptions. SRY may translocate from the Y to X chromosome during paternal meiosis (66, 67), an XY cell line may lurk in gonadal tissue, or a mutation or copy number variations in critical genes may upset the fragile equilibrium between protestis and proovary genetic pathways [reviewed in (68)]. Serum AMH above the low levels expected for a female sound the alert if the phenotype is ambiguous (Figure 4E).

**XX males** are endowed with bilateral testes and are usually fully virilized (Figure 4B); in most, but not all cases, Y material can be detected in their DNA, usually located on an X chromosome, but a minority of XX males are SRY negative and often sexually ambiguous. XX males resemble Klinefelter boys insofar as childhood is usually uneventful, XX germ cell degeneration sets in at puberty, and infertility is inevitable. AMH is in the normal range during childhood and falls subsequently (6 in Figure 6).

**Ovotesticular DSD** is a multifaceted condition defined by the coexistence in the same individual of testicular and ovarian tissue, either separate or associated within an ovotestis. External genitalia can be more or less virilized, but internally, a uterus is present in 70% of cases (69) and ovarian tissue is functional whereas testicular tissue is usually dysgenetic. An AMH serum level above normal female values in a sexually ambiguous XX baby is a strong argument for the diagnosis (7 in Figure 6). Values approaching male standards are found only during the first 2 weeks after birth in patients with large amounts of testicular tissue (35); later, AMH concentration decreases in keeping with the progressive degeneration of testicular tissue (69). Most karyotypes are 46,XX or 46,XX/46,XY chimerisms (Figure 4E). A 45,X/46,XY mosaic should awaken suspicion of mixed or asymmetrical gonadal dysgenesis, in case an undifferentiated streak with “ovarian stroma” has been mistaken for genuine ovarian tissue (70).

### 46,XX DSD Without Testicular Tissue

In this context, the androgens responsible for virilization of the external genitalia come from an extra-testicular, source but in the absence of Sertoli cells, AMH is not produced. **Congenital adrenal hyperplasia** (CAH) due to 21-hydroxylase deficiency accounts for more than half the cases of DSD and is the first diagnosis evoked by a pediatrician confronted with a sexually ambiguous or apparently male baby with impalpable testes. **Aromatase deficiency**, a rare condition due to a mutation in placental cP450arom (71), should only be considered in an XX sexually ambiguous child once CAH has been ruled out, and AMH is in the female range, thus ruling out ovotesticular DSD. Maternal ovarian tumors, for instance hCG-dependent **luteomas**, are another rare cause of XX DSD (72). Exogenous androgen or progestin administration during pregnancy, as a cause of fetal virilization, is extremely rare nowadays (73). In all these conditions, AMH serum values are in the female range (5 in Figure 6), uterus, Fallopian tubes, and ovaries are normal.

## SEX Chromosomal DSD

Sex chromosomal DSD designate conditions where sex chromosomes are neither uniformly XX nor XY. Mosaicisms or chimeras with at least one Y-chromosome lineage are usually associated with DSD due to mixed or asymmetrical or ovotesticular dysgenesis. The AMH level in serum is grossly correlated with the amount of functioning testicular tissue present (35).

### Turner Syndrome

Women with Turner syndrome have different karyotypes, all of which lack X chromosomal material; a 45,X karyotype is present in 40–50% of cases, 45,X/46,XX in 15–25% (74). Patients experience accelerated loss of ovarian follicles starting in fetal life and inexorably leading to ovarian insufficiency and infertility at various ages (75, 76). Preservation of fertility may be feasible by cryopreservation of ovarian tissue before follicles have totally vanished. AMH assay, often considered a marker of ovarian reserve, is detectable in a fifth of Turner girls (8 in Figure 6), essentially those with a mosaic 46,XX cell line, indicating that follicles are still present and cryopreservation is still a viable option.

### Klinefelter Syndrome

Klinefelter syndrome is the most common form of hypogonadism in males. It is characterized by a supernumerary X chromosome; the classical 47,XXY karyotype is present in 90% of cases. Cryptorchidism and mild developmental disorders may alert to the diagnosis in childhood, but usually patients are referred in adulthood for infertility (77). During childhood, AMH serum level is in the normal range during childhood, decreasing to lower levels after puberty (78, 79), indicating that Sertoli cell function is not impaired before mid to late puberty (6 in Figure 6).

## Concluding Remarks

AMH is a reliable biomarker of testicular and ovarian function and is extremely useful for the differential diagnosis of DSD (Figure 6), especially when confronted with serum testosterone concentration. Sertoli cells of the testes produce high amounts of AMH from early fetal life until the onset of puberty, while granulosa cells of the primary and small antral ovarian follicles produce small amounts of AMH from late fetal life until menopause. In patients with DSD, serum AMH is useful for the following purposes: (a) detect the existence of testicular tissue; (b) determine the amount of functional Sertoli cells; (c) distinguish between congenital disorders affecting whole testicular differentiation (gonadal dysgenesis) and those affecting exclusively Leydig cells or Sertoli cells; (d) direct the diagnosis of PMDS to the study of the *AMH* or the *AMHR2* gene; (e) detect the existence of ovarian follicles and the possibility or preserving fertility in girls with Turner syndrome; and (f) assess Sertoli cell function in boys with Klinefelter syndrome.

## Author Contributions

All authors listed have made a substantial, direct and intellectual contribution to the work, and approved it for publication.

## Conflict of Interest

NJ and RR have received payments from INSERM derived from royalties paid by Beckman-Coulter-Immunotech to INSERM for the development of an AMH ELISA kit. RR has received honoraria from CONICET (Argentina) for technology services using the AMH ELISA. Beckman is not a funder and has no role whatsoever in our work.
